# Report of a rare case of tinea capitis favosa caused by *Trichophyton schoenleinii* in Iran

**DOI:** 10.22034/cmm.2025.345462.1695

**Published:** 2026-02-01

**Authors:** Leila Hosseini Pour, Seyed Jamal Hashemi, Zeinab Borjian Boroujeni, Ahmad Abolfathi, Muhammad Ibrahim Getso, Omid Raiesi

**Affiliations:** 1 Department of Medical Parasitology and Mycology, School of Public Health, Tehran University of Medical Sciences, Tehran, Iran; 2 Department of Medical Biotechnology, Molecular Biology Research Center, Baqiyatallah University of Medical Sciences, Tehran, Iran; 3 Department of Medical Microbiology and Parasitology, College of Health Sciences, Bayero University, PMB, 3011, Kano, Nigeria; 4 Medical Analysis Department, Faculty of Applied Sciences, Tishk International University, Erbil, Iraq; 5 Department of Parasitology, School of Allied Medical Sciences, Ilam University of Medical Sciences, Ilam, Iran; 6 Student Research Committee, Ilam University of Medical Sciences, Ilam, Iran

**Keywords:** Tinea capitis, Tinea Favosa, *Trichophyton schoenleinii*, Iran, Case report

## Abstract

**Background and Purpose:**

Favus, a severe form of tinea capitis, is primarily caused by *Trichophyton schoenleinii* and can lead to permanent scarring alopecia if untreated. Although its prevalence has declined globally, outbreaks persist in regions with socioeconomic challenges and immigrant populations.

**Case Presentation:**

We report an 8-year-old Afghan boy residing in Iran who presented with diffuse alopecia and erythematous, purulent scalp lesions. Direct microscopy, culture, and ITS-PCR confirmed infection with *T. schoenleinii*. The isolate exhibited high susceptibility to itraconazole, ketoconazole, and terbinafine, with reduced activity for fluconazole. Treatment with topical ketoconazole and oral itraconazole (5 mg/kg/day for six months) resulted in clinical resolution.

**Conclusion:**

Early recognition of favus, coupled with accurate identification and susceptibility-guided therapy, is crucial to prevent long-term complications. Surveillance of *T. schoenleinii* in vulnerable populations, including immigrants, remains vital to inform public health strategies and mitigate the risk of re-emergence.

## Introduction

Tinea capitis is an infection of the scalp and hair that occurs when dermatophytes invade the hair shaft, producing a wide range of clinical appearances. It remains the most frequently encountered superficial fungal infection among children who have not yet reached puberty[1]. Because it spreads easily from person to person, clusters of cases can arise in schools and other communal settings, including those in developed countries. When the disease presents with pustules, it may heal with scarring and irreversible hair loss, creating both cosmetic challenges and emotional stress for affected children and their families[2].

Both inflammatory and non-inflammatory variants are recognized; the pattern of affectation, the predominant species, and the prevalence vary with time and geography, partly due to human migration. In some low-resource settings, recent estimates suggest that as many as 40% of children may be affected[3]. Favus-a distinct form of tinea capitis- is caused predominantly by the anthropophilic species *Trichophyton schoenleinii*, which accounts for more than 95% of cases. Less commonly, other dermatophytes from anthropophilic, zoophilic, or geophilic sources, such as *T. violaceum*, *T. quinckeanum*, *T. verrucosum*, and *Microsporum gypseum*, have been implicated [4]. The introduction of griseofulvin in 1958 dramatically reduced the prevalence of some anthropophilic species such as *T. schoenleinii* and *Microsporum audouinii* in many regions [2]. Currently, favus is found mainly in parts of Africa-including Nigeria [5] and Ethiopia [6] as well as western China [7], and other areas where poverty, overcrowding, and poor nutrition remain common [2]. Cases of tinea capitis favosa caused by Trichophyton schoenleinii are shown in Table 1.

**Table 1:** Cases of tinea capitis favosa caused by *Trichophyton schoenleinii* in the literature

Author	Year	Country	Sex/Age	Number of cases	Ref.
Akbari et al.	2024	Iran	M/8	1	[8]
Takaliuang et al.	2022	Indonesia	F/19	1	[9]
Sadati et al.	2021-2022	Iran	M/15, F/14, M/22	3	[10]
IWASA et al.	2019	Japan	F/63	1	[11]
Tlamcani et al.	2016	Morocco	F/2	1	[1]
Ghadgepatil et al.	2015	India	F/70	1	[12]
Erkan et al.	2015	Turkey	F/20	1	[13]
Yavuz et al.	2015	Turkey	F/42	1	[14]
Hassan et al.	2013	India	F/60	1	[15]
Zaraa et al.	2011	Tunisia	F/73	1	[16]
Khaled et al.	2007	Tunisia	M/6	1	[17]
Niczyporuk et al.	2004	Poland	F/48, F/10	2	[18]
F Botterel et al.	2001	Senegal	F/15	1	[19]
Velho et al.	2001	Portugal	F/5	1	[20]
Matte et al.	1997	Brazil	F/22	1	[21]
Siddaramappa et al.	1991	South India	M	1	[22]
HANSELL et al.	1955	England	F/14, M/1, F/33, F/12, F/8, F/6, F/6	7	[23]

Unfortunately, the efficacy of griseofulvin has declined over the years, often requiring increased dosages and prolonged courses before a clinical response is achieved. As a result, it is no longer considered the preferred option for most superficial dermatophyte infections. Newer antifungal classes-such as allylamines, triazoles, terbinafine, and the echinocandins-typically act more rapidly, allow shorter treatment schedules, and may continue exerting fungicidal effects for weeks after therapy ends, thereby reducing both treatment duration and associated adverse effects[2]. Despite the clinical significance of infections caused by *T. schoenleinii*, data on how this species responds to modern antifungal agents remain scarce across different regions. In this context, we describe an uncommon case of favus due to *T. schoenleinii* and evaluate the in-vitro susceptibility profile of the isolates using the Clinical and Laboratory Standards Institute (CLSI) broth microdilution method.

## Case Presentation

We report an 8-year-old Afghan male student who was evaluated at the Mycology Laboratory of the School of Public Health, Tehran University of Medical Sciences, on 7th July 2024, He presented with widespread hair loss affecting the scalp, accompanied by scattered patchy erythematous and purulent lesions. His medical history was notable only for moderate-to-severe allergic symptoms; he reported no family history of comparable conditions and had not used corticosteroids, broad-spectrum antibiotics, or medications for chronic illness. The abnormalities were confined to the scalp, and no lesions were detected elsewhere on the body. Hair and skin scrapings from affected sites were examined directly with lactophenol cotton blue stain and 10% KOH, which supported the suspicion of a dermatophyte infection. Culture subsequently yielded colonies consistent with *Trichophyton schoenleinii* (Figure. 1).

**Figure 1:**
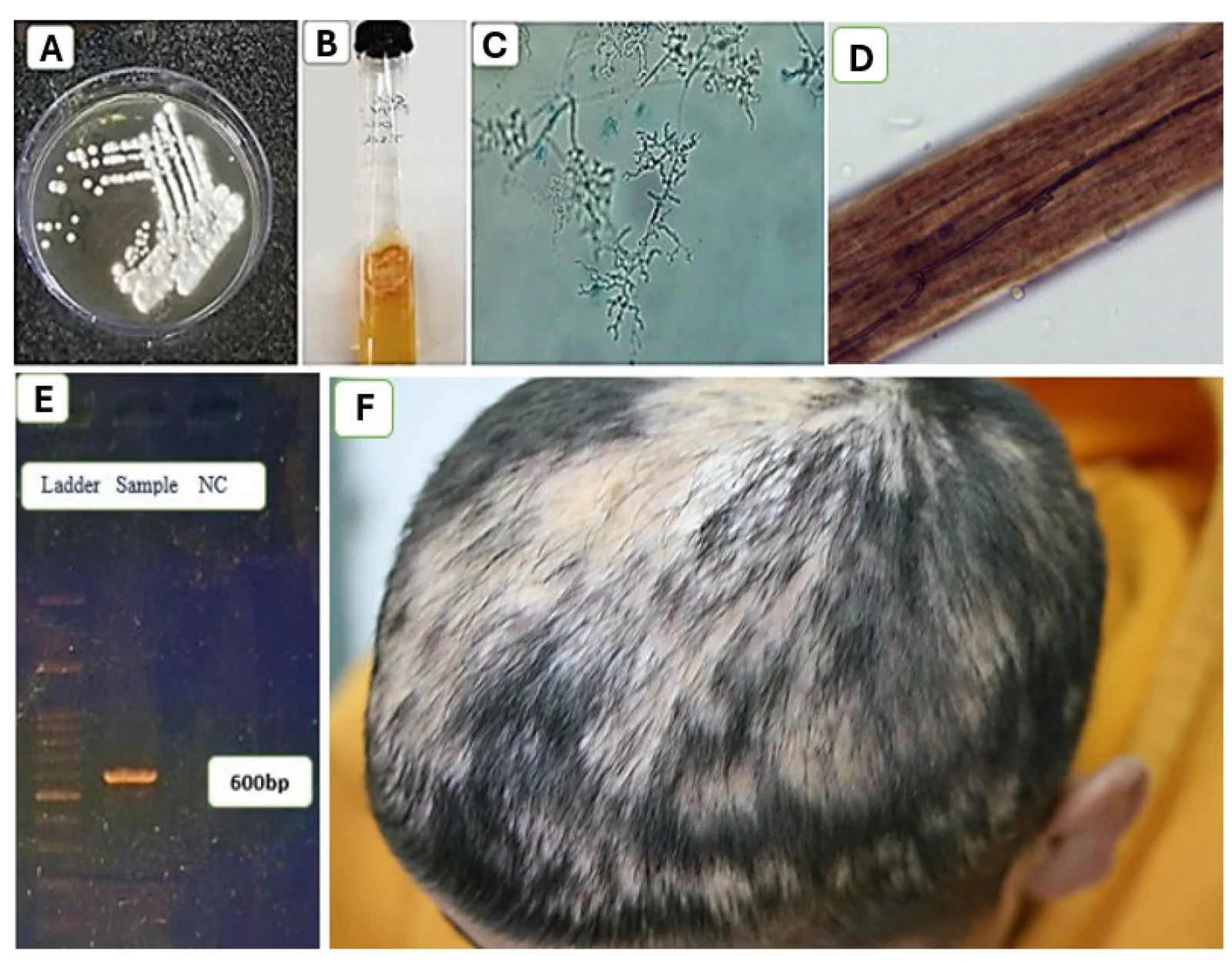
**A.** *Trichophyton schoenleinii* after 2 weeks of growth on Sabouraud Dextrose Agar with Chloramphenicol and Cycloheximide (SCC); **B.** Trichophyton schoenleinii after 4 weeks of growth in SCC; **C.** Knobby, antler-like hyphae with characteristic "favic chandelier" appearance in Lactophenol cotton blue stain; **D.** Diffuse erythematous lesions involving the scalp and hair with patchy alopecia

Molecular confirmation was performed using PCR targeting the Internal Transcribed Spacer (ITS) region of the ribosomal RNA gene. DNA was extracted from colonies grown on Sabouraud Dextrose Agar supplemented with chloramphenicol and cycloheximide by transferring the material into a 2-ml Eppendorf tube containing 400 µl of TEX buffer (Tris 1.2% w/v, Na-EDTA 0.38% w/v; pH 9.0) and glass beads, followed by vigorous mixing for 15 minutes on a MO-BIO vortex system. PCR amplification was carried out using standard denaturation (95 °C), annealing (56 °C), and extension (72 °C) cycles. The resulting product was sequenced by the Sanger method, and the ITS sequence was compared with entries in GenBank sequences through an NCBI BLAST search tool (https://blast.ncbi.nlm.nih.gov/Blast.cgi). The isolate showed 99% similarity to reference sequences of *T. schoenleinii*.

The sequence obtained in this investigation has been deposited in GenBank under the accession number PQ394801. Antifungal susceptibility was then assessed using the (CLSI M38-A) broth microdilution method[24]. Minimum inhibitory concentrations (MICs) of the clinical isolate for itraconazole (0.016 to 16 μg/ml), ketoconazole (0.016 to 16 μg/ml), fluconazole (0.064 to 64 μg/ml), terbinafine (0.0156 to 16 μg/ml), and griseofulvin (0.064 to 64 μg/ml) were 0.5, 0.25, 8, 0.12, and 0.5 μg/ml , respectively, indicating that the isolate was highly sensitive to these agents. Based on these findings, the patient was treated with oral itraconazole at 5 mg/kg/day for six months, topical ketoconazole (lotion and shampoo) together with oral itraconazole (5 mg/kg/day) for six months, resulting in a favorable clinical response.

## Discussion

Over the past several decades, the epidemiology of *Trichophyton schoenleinii*- the principal agent responsible for favus-has changed noticeably across Iran. Reports indicate that favus was historically more common in northern regions such as Rasht[25], where it accounted for 16.8% of dermatophytosis cases, compared with far lower rates in southern cities like Ahvaz, where only 2.3% of cases were attributed to this organism [26]. Although the basis for this regional contrast is not entirely clear, differences in reporting systems may contribute. Factors such as hygiene practices, household living conditions, socioeconomic disparities, and migration status all influence the distribution of dermatophyte infections.

Studies in Tehran showed that *T. schoenleinii* remains among the notable causes of tinea capitis, following *T. tonsurans*, *T. violaceum*, and *Microsporum canis* [4]. The continuing influx of immigrants from Afghanistan, combined with economic instability in some communities, may be contributing to the persistence of this infection in certain Iranian cities[8]. In contrast, the rarity of the disease in southern Iran can lead to diagnostic delays or even misdiagnosis, increasing the risk of complications. Because infections caused by this anthropophilic dermatophyte spread easily-particularly within households-any delay in recognition or treatment can lead to permanent scarring alopecia and associated psychosocial difficulties, as illustrated in the present case[10]. The rising number of *T. schoenleinii* isolates identified among Afghan immigrants in Iran represents an emerging public health concern and suggests the possibility of reintroduction of this infection into areas where it had become uncommon. Changing migration patterns, socioeconomic shifts, international travel, and unsupervised drug use all have the potential to alter the epidemiology of favus[8].

Continuous monitoring of dermatophyte species distribution is therefore essential. Identifying new cases and tracking species trends provide valuable data for refining public health policies and increasing public awareness of early symptoms, with the aim of reducing transmission [11].

Susceptibility assessments conducted by Deng et al. on 55 isolates of *T. schoenleinii* collected over three decades from Iran, Turkey, and China using the CLSI broth microdilution method showed that terbinafine and ketoconazole were the most potent antifungal agents regardless of the geographical origin of the strains. These findings align with earlier reports in which terbinafine exhibited MIC values between 0.02 and 0.13 μg/mL against dermatophytes isolated from tinea capitis cases. Except for fluconazole, the azoles generally demonstrated strong *in vitro* activity against *T. schoenleinii* [27]. Additional studies further support these observations. Fernández-Torres et al. evaluated two strains of *T. schoenleinii*, and found itraconazole, voriconazole, and miconazole to have low MIC ranges (0.01–0.05 μg/mL, 0.01–0.06 μg/mL, and 0.031–0.063 μg/mL, respectively), while fluconazole exceeded 16 μg/ml [28]. Another study conducted by Indira et al indicated that ketoconazole and itraconazole displayed MIC ranges of 0.06–0.96 mg/mL and 0.12–0.96 mg/mL. Several other studies likewise confirm the robust in-vitro efficacy of azole antifungals against this species. [29]. Several other studies likewise confirm the robust in-vitro efficacy of azole antifungals against this species [30]. Although griseofulvin was the recommended therapy for tinea capitis for nearly forty years, it is no longer favored for superficial dermatophytosis. Over time, its effectiveness has diminished, and strains with reduced susceptibility have been documented [2].

## Conclusion

In this study, we reported a rare case of tinea capitis favosa due to *T. schoenleinii* in an 8-year-old Afghan male student with symptoms of patchy alopecia that was successfully treated with topical ketoconazole (lotion and shampoo) and itraconazole (5 mg/kg/day). Terbinafine and several azole antifungals retain strong in-vitro activity against *T. schoenleinii*, whereas griseofulvin-historically the standard therapy-shows declining effectiveness. Continuous surveillance of dermatophyte species distribution, coupled with improved public health awareness, is essential for early detection, appropriate treatment selection, and the prevention of disease resurgence.

## References

[1] Tlamcani Z, Figuigui S, Taghouti A (2016). A case of Tinea capitis favosa due to Trichophyton schoenleinii in Morocco. Sch J App Med Sci.

[2] Shemer A, Plotnik IB, Davidovici B, Grunwald MH, Magun R, Amichai B (2013). Treatment of tinea capitis–griseofulvin versus fluconazole–a comparative study. JDDG.

[3] Aharaz A, Jemec G, Hay R, Saunte D (2021). Tinea capitis asymptomatic carriers: what is the evidence behind treatment?. J Eur Acad Dermatol Venereol.

[4] Bassiri-Jahromi S, Khaksari AA (2009). Epidemiological survey of dermatophytosis in Tehran, Iran, from 2000 to 2005. Indian J Dermatol Venereol Leprol.

[5] Nweze EI (2001). Etiology of dermatophytoses amongst children in northeastern Nigeria. Med Mycol.

[6] Figueroa JI, Hawranek T, Abraha A, Hay RJ (1997). Tinea capitis in south-western Ethiopia: a study of risk factors for infection and carriage. Int J Dermatol.

[7] Deng S, Bulmer GS, Summerbell RC, De Hoog GS, Hui Y, Graser Y (2008). Changes in frequency of agents of tinea capitis in school children from Western China suggest slow migration rates in dermatophytes. Med Mycol.

[8] Akbari N, Parhiz J, Sabzi Z (2024). Tinea capitis (Favus) in a 8-year-old child: Case report. Clin Case Rep.

[9] Takaliuang DS (2022). Tinea capitis in adolescent: A case report. Eduvest J Univ Stud.

[10] Handjani F, Zomorodian K, Pakshir K, Geramoshoar M, Radanfar R (2023). Tinea favosa: negligence and misdiagnosis in three cases in Shiraz during 2021-2022. Jundishapur J Health Sc.

[11] Iwasa K, Ogawa K, Azukizawa H, Tanabe H, Iwanaga T, Anzawa K (2019). Revival of favus in Japan caused by Trichophyton schoenleinii. J Dermatol.

[12] Ghadgepatil SS, Sharma YK, Misra R, Dash KN, Patvekar MA, Deo KS (2015). An unusual case of tinea capitis caused by Trichophyton schoenleinii in an elderly female. Indian Dermatol Online J.

[13] Erkan D, Kolukirik I, Acar A, Kandemir H, Ilkit M (2015). [A recurrent case of adult favus successfully treated with terbinafine].. Mikrobiyol Bul.

[14] Özaydın Yavuz G, Yavuz İH (2015). Favus: A case report. Van Med J.

[15] Hassan I, Rather PA, Sajad P (2013). Favus in an elderly Kashmiri female: A rare occurrence. Indian J Dermatol.

[16] Zaraa I, Zaouek A, El Euch D, Trojjet S, Mokni M, Ben Osman A (2012). Tinea capitis favosa in a 73-year-old immunocompetent Tunisian woman. Mycoses.

[17] Khaled A, Mbarek L, Kharfi M, Zeglaoui F, Bouratbine A, Fazaa B (2007). Tinea capitis favosa due to Trichophyton schoenleinii. Acta dermatovenerologica alpina panonica et adriatica.

[18] Niczyporuk W, Krajewska-Kulak E, Lukaszuk C (2004). Tinea capitis favosa in Poland. Mycoses.

[19] Botterel F, Romand S, Cornet M, Recanati G, Dupont B, Bouree P (2001). Dermatophyte pseudomycetoma of the scalp: case report and review. Br J Dermatol.

[20] Velho GD, Selores M, Amorim I, Lopes V (2001). Guess what! Tinea capitis by Trichophyton schoenleinii. Eur J Dermatol.

[21] Matte SM, Lopes JO, Melo IS, Beber AA (1997). A focus of favus due to Trichophyton schoenleinii in Rio Grande do Sul, Brasil. Rev Inst Med Trop Sao Paulo.

[22] Siddaramappa B, Hemashettar M, Patil S (1991). Favus from South India-A case report. Indian J Dermatol Venereol Leprol.

[23] Hansell J, Partridge BM (1955). Favus: a report of seven related cases. Br Med J.

[24] CLSI (2008). Reference method for broth dilution antifungal susceptibility testing of filamentous fungi; approved standard, 2nd ed CLSI document M38-A2, vol 28, no. 16.

[25] Alizadeh N, Sadr Ashkevary S, Golchai J, Maboodi A, Falahati A (2004). Descriptive study of dermatophytosis in Guilan. Iranian J Dermatol.

[26] Rezaei-Matehkolaei A, Rafiei A, Makimura K, Graser Y, Gharghani M, Sadeghi-Nejad B (2016). Epidemiological aspects of dermatophytosis in Khuzestan, southwestern Iran, an update. Mycopathologia.

[27] Deng S, Ansari S, Ilkit M, Rafati H, Hedayati MT, Taghizadeh-Armaki M (2017). In Vitro antifungal susceptibility profiles of 12 antifungal drugs against 55 Trichophyton schoenleinii isolates from tinea capitis favosa patients in Iran, Turkey, and China. Antimicrob Agents Chemother.

[28] Fernández-Torres B, Carrillo A, Martın E, Del Palacio A, Moore M, Valverde A (2001). In vitro activities of 10 antifungal drugs against 508 dermatophyte strains. Antimicrob Agents Chemother.

[29] Indira G (2014). In vitro antifungal susceptibility testing of 5 antifungal agents against dermatophytic species by CLSI (M38-A) micro dilution method. Clin Microbial.

[30] Girgis SA, El-Fakkar N, Bard H, Shaker OA, Metwally FE, Bassim HH (2006). Genotypic identification and antifungal susceptibility pattern of dermatophytes isolated from clinical specimens of dermatophytosis in Egyptian patients. Egyptian Dermatol Online J.

